# Face Mask Identification Using Spatial and Frequency Features in Depth Image from Time-of-Flight Camera

**DOI:** 10.3390/s23031596

**Published:** 2023-02-01

**Authors:** Xiaoyan Wang, Tianxu Xu, Dong An, Lei Sun, Qiang Wang, Zhongqi Pan, Yang Yue

**Affiliations:** 1 Institute of Modern Optics, Nankai University, Tianjin 300350, China; 2 National Center for International Joint Research of Electronic Materials and Systems, School of Electrical and Information Engineering, Zhengzhou University, Zhengzhou 450001, China; 3 Shphotonics, LLC, Tianjin 300450, China; 4 Angle AI (Tianjin) Technology Co., Ltd., Tianjin 300450, China; 5 Department of Electrical & Computer Engineering, University of Louisiana at Lafayette, Lafayette, LA 70504, USA; 6 School of Information and Communications Engineering, Xi’an Jiaotong University, Xi’an 710049, China

**Keywords:** 3D data processing, depth camera, face mask identification

## Abstract

Face masks can effectively prevent the spread of viruses. It is necessary to determine the wearing condition of masks in various locations, such as traffic stations, hospitals, and other places with a risk of infection. Therefore, achieving fast and accurate identification in different application scenarios is an urgent problem to be solved. Contactless mask recognition can avoid the waste of human resources and the risk of exposure. We propose a novel method for face mask recognition, which is demonstrated using the spatial and frequency features from the 3D information. A ToF camera with a simple system and robust data are used to capture the depth images. The facial contour of the depth image is extracted accurately by the designed method, which can reduce the dimension of the depth data to improve the recognition speed. Additionally, the classification process is further divided into two parts. The wearing condition of the mask is first identified by features extracted from the facial contour. The types of masks are then classified by new features extracted from the spatial and frequency curves. With appropriate thresholds and a voting method, the total recall accuracy of the proposed algorithm can achieve 96.21%. Especially, the recall accuracy for images without mask can reach 99.21%.

## 1. Introduction

Face masks are a well-established preventive tool to limit the spread of viruses through droplets and aerosols in the population [1]. With the COVID-19 global pandemic, many countries and regions stipulate that people must wear masks in public places, especially indoor areas and vehicles. In most cases, some staff are assigned to enforce the rule. Nevertheless, the manual monitoring method not only wastes manpower but also can easily cause infection. Contactless mask recognition can avoid the waste of human resources and the risk of exposure, which has been highly valued by many researchers [2,3,4].

It is important to quickly and accurately identify masks in daily life, especially in areas with a large flow of people. Some researchers have developed integrated systems for rapid screening. Hussain et al. [5] designed an intelligent disinfection screening door that can simultaneously measure the pedestrian’s temperature, identify if a mask is worn, and perform the disinfection. The 2D data captured by the camera was first input to the mask recognition module. Then, the neural network was used for classification. In addition to the comprehensive screening system, which can monitor the temperature and detect the mask, there is also a lot of work focused on improving the accuracy of mask recognition. Most mask recognition methods are based on object recognition technology, which automatically identifies whether a person is wearing a mask by combining the camera’s input with a computer algorithm. Conventional solutions of object recognition are mainly based on two-dimensional (2D) images, including detecting regions of interest, extracting features, and performing classification [6]. At present, numerous emerging machine learning methods are being used more and more in object recognition. In [7,8,9,10,11,12], ResNet, Yolo, MobileNet, and other machine learning models are used to recognize face masks. The identification accuracy can exceed 90%. However, the performance of these methods begins to decline significantly in some situations, such as occlusion or variable illumination conditions [13]. In addition, Cao et al. [14] considered night situations and proposed the MaskHunter model, but the accuracy dropped to 71.6%. Even when the additional training module was designed to improve accuracy, the results were not satisfactory. Hence, breaking the trade-offs between accuracy and the adaptability of mask identification is a difficult problem to solve.

In recent years, with the enrichment of information acquisition methods and the development of sensing technology, many three-dimensional (3D) sensors have emerged [15,16], which can not only obtain the shape information of the object but also determine the distance between the object and the camera. Among them, the depth camera, based on the principle of active ranging, has many advantages, such as being insensitive to illumination and unaffected by the contrast of the target. As the popular depth sensors that are commercially available [17,18,19], the structured light cameras and the time-of-flight (ToF) cameras have different advantages and application scenarios [20]. In most cases, the ToF camera has low complexity and advantages in practical application [21]. Therefore, the ToF camera is more commonly used as a data acquisition device. Since the depth cameras work well in low light and even in dark conditions, object recognition based on obtained 3D images has been used in many scenes, such as human detection, industrial assembly, gesture recognition, and others [22,23,24,25,26,27,28]. Luna et al. [29] presented a new method for detecting people only using depth images, and the data was captured by a depth camera in a frontal position. This method ran in real-time using a low-cost CPU platform with high accuracy. Various tasks based on the depth information require different features to be extracted. The introduction of 3D information also increases the computational cost of machine learning models. Moreover, the universal parameters of feature extraction based on 3D information need to be further investigated.

In this paper, we propose and demonstrate a method based on a ToF depth camera to determine whether a person is wearing a mask. The results are divided into three classifications: wearing no mask, wearing a surgical mask, and wearing an N95 mask. The three situations can be easily discriminated by the optimized spatial and frequency features on the facial depth contour. The experimental results show that these characteristics not only can distinguish whether people wear masks but also can determine the types of masks. Unlike the 2D image from the RGB camera, which is easily affected by the environmental illumination, our developed mask identification system based on the ToF camera runs robustly under variable external conditions, especially in the dark environment. Compared with the other 3D imaging sensors, the ToF camera also has the advantage of fast imaging. Through the sampling and dimension reduction of the depth image to obtain the facial contour curve, our method could potentially achieve quick and accurate identification, which is suitable for scenes with illumination variation and rapid identification, such as entrances and exits of the building. In addition, our method can also provide statistical data for epidemiological analysis by monitoring the mask types.

## 2. Method

**Figure 1:**
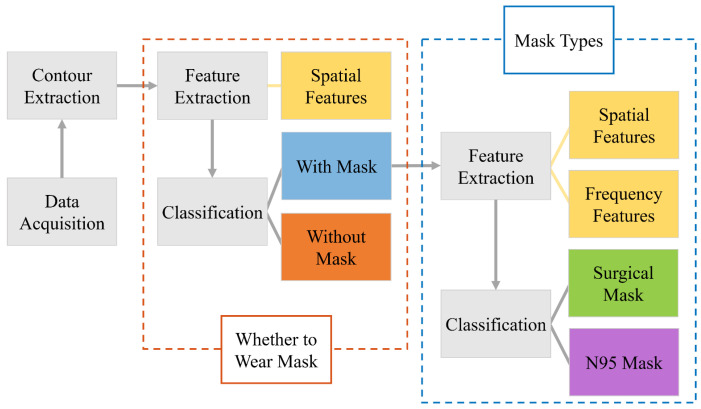
Flow chart of mask recognition based on depth camera.

Figure 1 illustrates the process of the proposed mask recognition method. In the data acquisition module, the ToF camera is used to collect the facial depth images. The facial contour is then extracted from the depth image as a recognition feature in the next step. The recognition module can be sequentially divided into two sub-modules: one first needs to identify whether a person is wearing a mask. If the output result is yes, the mask type is further differentiated. Otherwise, the module can export the result that there is no mask. The above classification process mainly relies on the feature descriptor, including the spatial and frequency features. The spatial features are extracted from the facial contour, and the frequency features are obtained from the frequency characteristics by using the Fourier transform. There are two popular mask types verified by this method: surgical masks and N95 masks. Surgical masks are the most widely used in daily life, and N95 masks have better prevention abilities and a higher usage rate in hospitals and other risk environments [30]. Thus, the proposed method has great research significance for identifying these two mask types.

### 2.1. Facial Contour Extraction

In order to process the facial data efficiently and save on computational costs, the vertical facial contours are extracted from the 3D facial depth images to achieve rapid recognition. In sequence, spatial features and frequency features are determined from the extracted facial contour. Thus, the precision of the facial contour extraction directly affects the accuracy of the classification. In our method, a contour extraction approach is utilized to obtain appropriate facial contours. In addition to the facial information, there is a lot of redundant information on the obtained depth images. Those useless backgrounds need to be first removed by the clipping method. The designed rhombus template is then applied to reduce the interference of the hair and other noise. The facial contour is ultimately extracted from the rhombic region. The extracted facial contour is smoothed to obtain a contour curve with spatial interpretation.

**Figure 2:**
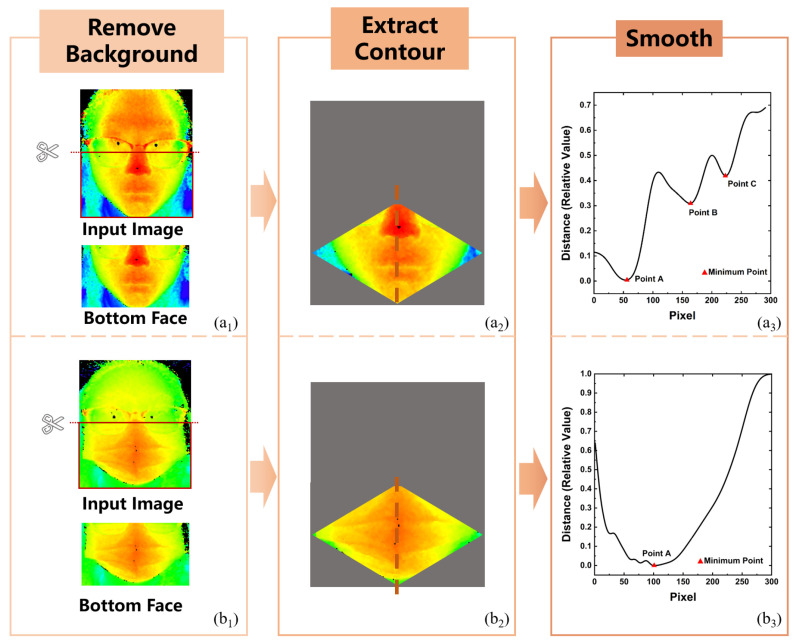
Overview of facial contour extraction. (**a**) Facial contour extraction of the image without a mask and (**b**) facial contour extraction of the image with a surgical mask.

The above-mentioned process and visualization results of contour extraction are shown in Figure 2. To avoid the influence of background information in the depth image, the human facial depth image is first segmented by the distance filter. With the distance information between the object and the camera being recorded in the depth image, the human face can be easily distinguished from other backgrounds by conditional filtering. By setting the segmented ranges of distance, one can highlight the foreground human face by performing a one-time filtering operation. The camera we used can capture complete and clear faces within the range from 0.1 m to 0.5 m. Therefore, the background with a distance greater than 0.5 m is filtered. The background and face can be completely separated by distance filtering. The images containing only the face can be obtained by setting the background pixel value to infinite and cutting out the pixels with the infinite value. Since the main difference between the images with and without masks is in the bottom part of the face, we directly cut out the top face in the image and only focus on the bottom face with obvious features. As shown in the first column of Figure 2, the whole input image is fully occupied by the face, both horizontally and vertically. Thus, the bottom face can be obtained by clipping from the middle of the image.

In the extraction process of human facial contour, a rhombus template is designed to filter the redundant hair noise. Through the above steps, we can obtain the bottom face in Figure 2, and the construction of the rhombus is based on the bottom face image. The midpoint of each side in the bottom face image is first picked out. These four points are then taken as the vertices of the rhombus. As shown in the second column of Figure 2, the overlapping part of the rectangular image and rhombus is the target area, and the rest of the rectangular image is removed. The gray part in the figure represents all the filtered parts of the original image, and the contour curve is extracted from the unfiltered color part. According to the geometric characteristics of the human face and depth distance, the facial contour can be obtained by finding the points with the minimum distance in each horizontal line of the rhombus area. The face contour is used to replace the original data, and the feature descriptor is further extracted, which greatly reduces the amount of calculation.

Due to the limited resolution of the depth camera, the extracted facial contour needs to be smoothed so that it can be closer to the original appearance. The extracted facial contour is smoothed with a Gaussian-weighted moving average filter, and the length of the smoothing window is 50. The example of contour smoothness is demonstrated in the third column of Figure 2. The ordinate of the curve is the relative distance between the face and the camera, and the abscissa is the pixel position. The first row (a) displays the contour extraction process of the images without a mask. As can be seen from the curve of a_3_, there are three obvious minimum points, A, B, and C, representing the tip of the nose, the top lip, and the bottom lip, respectively. The second row (b) illustrates the contour extraction process of the image with a surgical mask. For contour curves with a mask, point A only represents the global minimum point of the curve, which is caused by the mask shape instead of the nose. Other extreme points of the curve are caused by the mask wrinkles, which have no special significance and are therefore not annotated. All the contour curves need to be normalized, including unifying the length and setting the relative distance of the minimum point to zero.

### 2.2. Feature Extraction and Classification of Whether to Wear Mask

**Figure 3:**
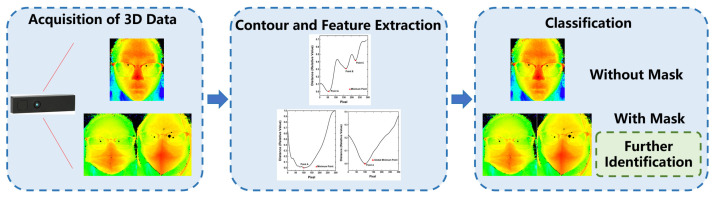
Identification process for whether or not to wear a mask.

After obtaining the facial contour curve, the first classification step is deciding whether to wear a mask. The process is shown in Figure 3, and this step identifies wearing or not wearing a mask. If the classification result is with a mask, it is necessary to further identify the mask type. The features used in this classification will be introduced in the following content. Three features are designed from the contour curve to facilitate the classification. The main features are the number of local minimum points on the contour curve, the standard deviation between the farthest points among the local minimum points, and the quadratic coefficient of the expression *Y_1_ = a_1_x^2^ + b_1_x + c_1_*, which is used to partially fit the contour curve.

**Figure 4:**
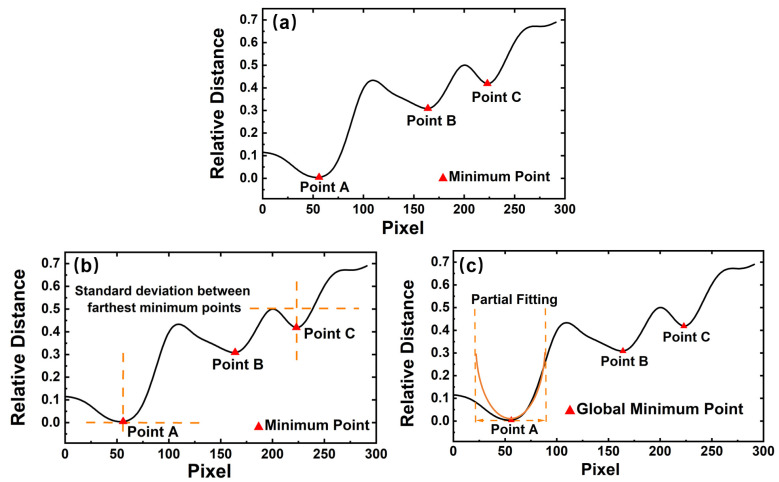
Three features of the image without a mask: (**a**) number of minimum points of the contour curve; (**b**) standard deviation between the farthest minimum points of the contour curve; and (**c**) quadratic coefficient of the partial fitting curve.

Figure 4 depicts the above features of the contour curve without a mask. The local minimum points of the contour curve are labeled in Figure 4a, where point A is the tip of the nose, point B represents the top lip, and point C is the bottom lip. The local minimum points are calculated according to Equation (1):

(1) $$\left\{\begin{array}{c} f\left(x_{j}\right)<f\left(x_{j-1}\right)\\ f\left(x_{j}\right)<f\left(x_{j+1}\right) \end{array}\right.,$$

where *f(x_j_)* is the depth value corresponding to point *x_j_*, *x* is the random point of the contour curve, and the range of *j* is from 2 to the length of contour curve. When *x_j_* satisfies Equation (1), it is recorded as the local minimum point of the curve. The number of the local minimum point is represented by *N.*

The standard deviation of the two farthest minimum points is denoted by *SSD* (spatial standard deviation). Point A and point C are the farthest minimum points shown in Figure 4b. *SSD* is calculated as follows:

(2) $$SSD=\sqrt{\frac{\sum_{j=1}^{n_{s}}{\left(x_{j}-\bar{x}\right)}^{2}}{n_{s}-1}},$$

where *n_s_* is the number of points between point A and point C, $\bar{x}$ is the average of these points. Especially, when the contour curve has only one minimum point, the value of *SSD* is 0.

Figure 4c explains the partial fitting process. Taking the global minimum of point A of the curve as the center, and the segment curve with the length of 2*L* is then obtained by taking *L* length from both sides of the curve. The quadratic function *Y*_1_ = *a*_1_*x*^2^ + *b*_1_x + *c*_1_ is used to fit this segment curve. According to the properties of the quadratic function, the quadratic coefficient *a*_1_ determines the opening size of the parabola. The value of |*a*_1_| is larger and the opening size of the parabola is smaller.

After calculating the above features, the type of contour curve can be determined according to the different feature values. Generally, the feature value of the curve without a mask are greater than the feature value of the curve with a mask. Therefore, the threshold values can be set to separate them. For the images without masks, *N* > *N’, SSD* > *SSD’, a*_1_ > *a*_1_’, where *N’*, *SSD’,* and *a*_1_’ are the thresholds. The voting method is used as classifier to determine whether a mask is worn or not. When more than two of the three features meet the threshold, it is determined that the mask is not worn.

### 2.3. Feature Extraction and Classification of Mask Types

**Figure 5:**
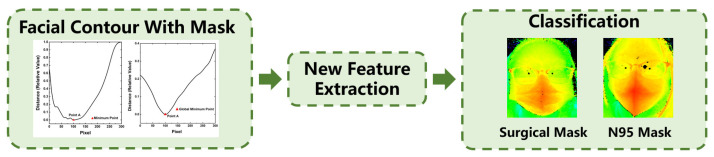
Identification process for mask types.

After identifying that a mask is worn, a further judgement of the mask type is executed. The judgement process is similar to identifying whether to wear a mask or not, which is shown in Figure 5. In our experiment, the mask types are the surgical mask and the N95 mask, respectively. The judgement process is similar to identifying whether to wear a mask. To obtain suitable features to recognize the mask types, three new features are designed from the spatial and frequency levels, including the opening angle of the contour curve, the quadratic coefficient of the expression *Y*_1_ = *a*_1_*x*^2^ + *b*_1_x + *c*_1_(which is used to partially fit the contour curve), and the area of the frequency curve. The corresponding frequency curve is obtained by the Fourier transform.

**Figure 6:**
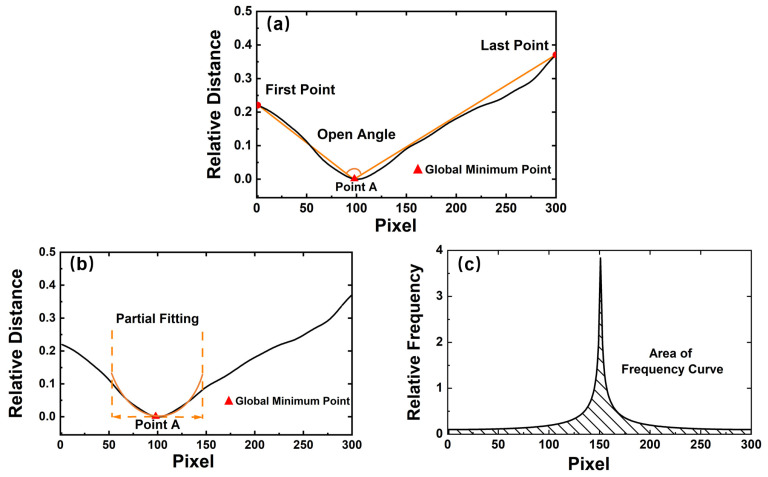
Three features of N95 mask: (**a**) opening angle of the contour curve, (**b**) quadratic coefficient of the partial fitting curve, and (**c**) area of the frequency curve from the Fourier transform of the spatial contour curve.

Figure 6 depicts the feature visualization of the contour curve with the N95 mask being worn. For contour curves with a mask, point A only represents the global minimum point of the curve, which is caused by the shape of the mask instead of the nose. The global minimum point (*x_M_*, *y_M_*), the left-end point (*x_L_*, *y_L_*), and the right-end point (*x_R_*, *y_R_*) of the contour curve are first located, as shown in Figure 6a. The opening angle, denoted by α, is computed from these three points. Taking the global minimum point as the vertex, the angle between two straight lines is calculated as follows:

(3) $$\alpha =arctan(\left|\frac{\frac{y_{M}-y_{L}}{x_{M}-x_{L}}-\frac{y_{R}-y_{M}}{x_{R}-x_{M}}}{1+\left(\frac{y_{M}-y_{L}}{x_{M}-x_{L}}\right)\cdot \left(\frac{y_{R}-y_{M}}{x_{R}-x_{M}}\right)}\right|)$$

The process of partial fitting explained in Figure 6b is consistent with Section 2.2. The quadratic function *Y*_1_ = *a*_1_*x*^2^ + *b*_1_x + *c*_1_ is used to fit the segment curve. According to the properties of the quadratic function, the quadratic coefficient a1 can also distinguish between the mask types.

After fitting the contour curves with the quadratic function *Y*_2_ = *a*_2_*x*^2^ + *b*_2_*x* + *c*_2_, its frequency curve is obtained by the Fourier transform in Figure 6c. The Fourier transform of the discrete contour curve follows Equation (4):

(4) $$\left\{\begin{array}{c} F\left(K\right)=\overset{n}{\underset{j=1}{\sum }}x\left(j\right)W_{n}^{\left(j-1\right)\left(k-1\right)}\\ W_{n}=e^{\frac{-2\pi i}{n}} \end{array}\right.,$$

where *F(K)* is the frequency curve from the Fourier transform, *n* is the number of the point on the contour curve, and *Wn* is a *n*-th root of unity. The area of the frequency curve is denoted by *FS* and it is calculated as follows:

(5) $$FS=\frac{1}{2}\overset{n}{\underset{j=1}{\sum }}\left(f\left(x_{j}\right)+f\left(x_{j+1}\right)\right)$$

Due to the different geometric shapes, two mask types have distinct feature values and the appropriate thresholds can separate the mask types. For surgical masks, *α < α’, a*_1_ *< a*_1_*’, FS* < *FS’*, where *α’, a*_1_*’,* and *FS’* are the thresholds. The voting method is also used to determine the mask types. When two or more of the above thresholds are met, the mask type is determined to be the surgical mask. Otherwise, the type is the N95 mask.

**Figure 7:**
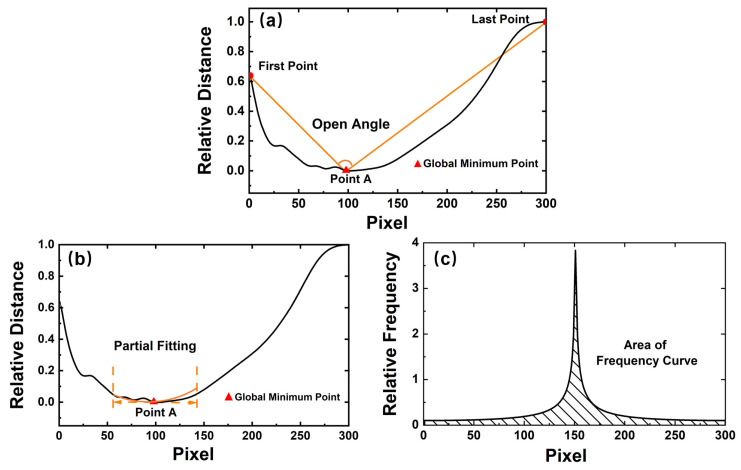
Three features of surgical masks: (**a**) opening angle of the contour curve, (**b**) quadratic coefficient of the partial fitting curve, and (**c**) area of the frequency curve from the Fourier transform of the spatial contour curve.

Figure 7 depicts the feature visualization of the contour curve with the surgical mask being worn. Compared to Figure 6, the partial fitting curve is quite different, and a clearer comparison will be analyzed in the next section.

## 3. Results

**Figure 8:**
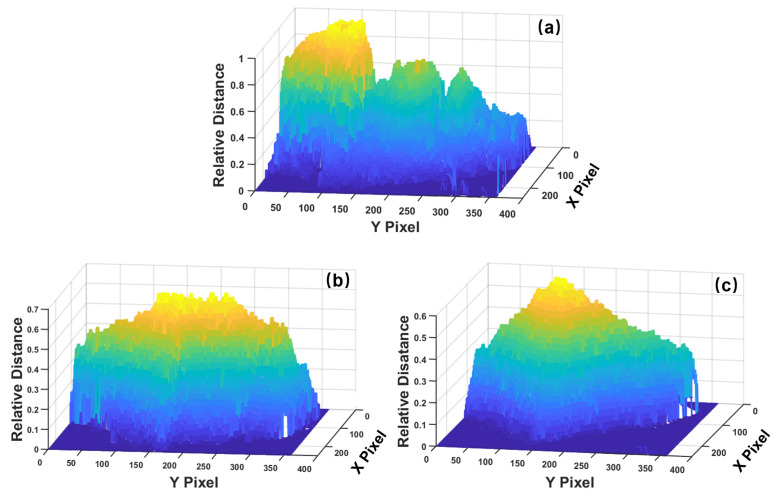
3D view of the bottom face acquired from the ToF camera: (**a**) without a mask, (**b**) with a surgical mask, and (**c**) with an N95 mask.

To evaluate the proposed recognition algorithm, a ToF depth camera was used to capture the depth data. The PicoFlexx camera from pmdtechnologies was used for data collection. The PicoFlexx camera is equipped with an IRS1145C Infineon^®^ REAL3™ 3D image sensor based on the principle of ToF. The camera uses an 850-nm Vertical-Cavity Surface-Emitting Laser (VCSEL) as the light source, and the detection range is from 0.1 m to 4 m. We used this camera to collect the front-face image under the indoor environment. For the acquired images, the corresponding resolution is 945 × 722 pixels, which is expanded from the 244 × 172 resolution of the IRS1145C sensor by the Software Development Kit (SDK) Royale 3.20.0.62 as a color-coded depth map. These depth images can be divided into three categories: not wearing a mask, wearing a surgical mask, and wearing an N95 mask. The bottom half of the face is obtained by distance filtering and clipping. The 3D view of three situations is shown in Figure 8. The pixel value of the data is normalized to the range from 0 to 1 by the SDK of the ToF camera, where 0 represents the closest distance to the camera and 1 represents the farthest distance detected by the camera. In Figure 8, we flip the meaning of the values for better visualization.

From the 3D view of the bottom face, the face without a mask has more obvious undulations with multiple peaks, and the nose part is conspicuous. The 3D contour of the surgical mask is much smoother in general, but there are more small fluctuations. The shape of the 3D contour for the N95 mask has only one obvious peak.

**Figure 9:**
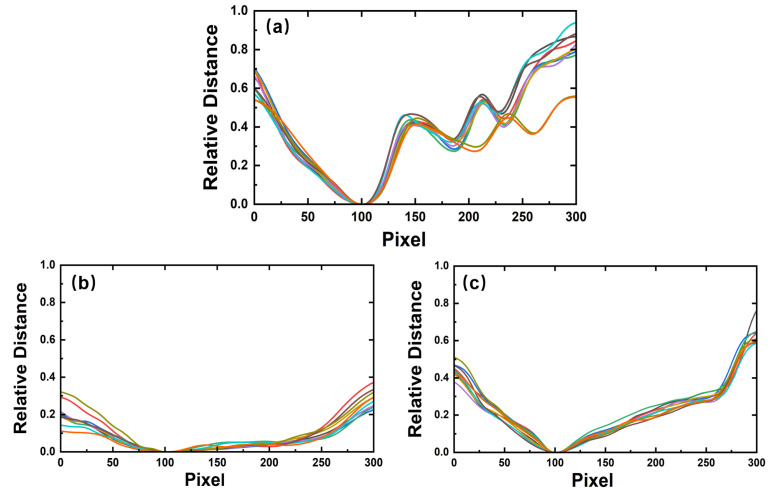
Normalized facial contour curves of 10 samples: (**a**) facial contours without a mask, (**b**) facial contours with a surgical mask, and (**c**) facial contours with an N95 mask.

With the depth image of the bottom face, the facial contour curve is extracted and normalized. In our research, the length of all the contour curves is unified at 300, and the relative distance of the global minimum point is set to zero. Figure 9 shows the 3 types of facial contour curves, and 10 contour curves of each type are selected for overlapping display. When all the contours are normalized, contour curves of the same type show good repeatability and similar geometric features, but different kinds of contour curves have different geometric features. In particular, the mask covers the shape of the human face, so the contour curve with a mask is gentler, while the contour curve without a mask fluctuates more obviously. We can clearly see the characteristics of the bottom face in Figure 9, including the nose, the top lip, and the bottom lip, while they cannot be distinguished from the contour curve with a mask. The contour curves of different mask types are also different. In general, the contour curve of a surgical mask is relatively gentle. However, there are many wrinkle-caused fluctuations in the curve. The N95 mask is made of thicker material, and its depth contour curve is closer to a trapezoidal shape. Depending on the different shapes of the contour curves, the features can be extracted for classification and recognition.

**Figure 10:**
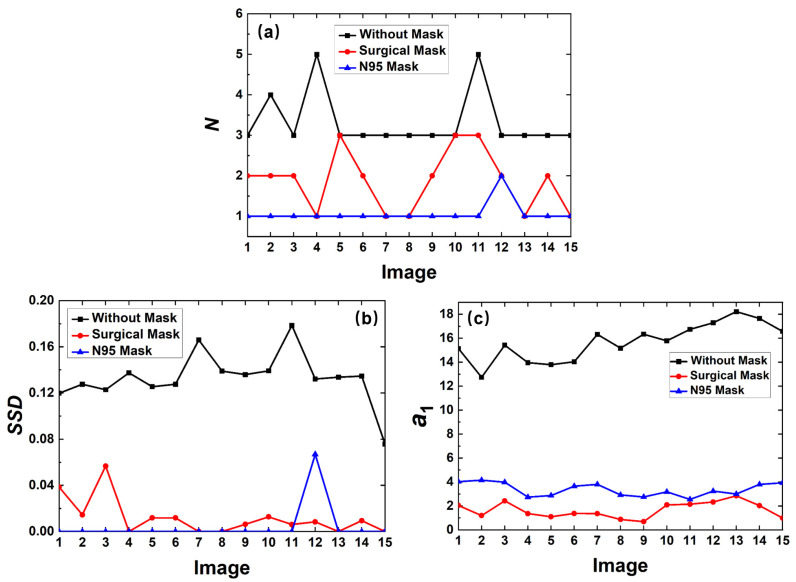
Feature values to identify whether to wear mask captured from 15 images: (**a**) the *N* value, (**b**) the *SSD* value, and (**c**) the *a*_1_ value of three types of curves.

After obtaining the contour curve, the features *N*, *SSD,* and a_1_ are calculated to determine whether to wear a mask. A total of 15 images of each type are selected to calculate the three features, which are shown in Figure 10. The black curve represents the feature values of the contour curve without a mask, while the red and blue curves represent the feature values of the contour curve with a surgical mask and an N95 mask, respectively. The minimum number of points is displayed in Figure 10a. One can see that due to the shape of the nose and lips, the number of the minimum points of the contour curve without a mask is always more than three. With the regular shape, the *N* value of the contour curve with an N95 mask is typically one. However, due to the influence of the folds, the *N* value of the contour curve with a surgical mask shows great fluctuation, ranging from 1 to 3. Although most *N* values of the contour curve without mask are greater than those of the contour curves with a mask, there are still overlapping parts. Therefore, a single feature cannot classify the mask wearing condition accurately. The value of *N* is also not suitable for distinguishing the mask types.

Figure 10b shows the standard deviation between the two farthest minimum points. The *SSD* values of the contour curve without a mask are far greater than those of the contour curve with a mask because of the shape of the human face. The folds of the surgical mask lead to the oscillation of the contour curve with the mask, causing a smaller but unpredictable fluctuation in *SSD* value. The contour curve with the N95 mask usually has only 1 minimum point, and its *SSD* is set to 0. However, because the *SSD* curves of two types of masks also overlap, the *SSD* value cannot distinguish the mask types well.

Figure 10c demonstrates the quadratic coefficient *a*_1_ of the partial fitting curve. As one can see, the a_1_ value of the contour curve without a mask are far greater than those of the contour curve with a mask. For the quadratic function, the value of |*a*_1_| is larger and the opening of the parabola is smaller. The global minimum point is the vertex of the quadratic function. For the contour curve without a mask, the fitting curve represents the nose. For the contour curve with a mask, the fitting curve describes a part of the mask. Therefore, the *a*_1_ value of the contour curve without a mask is greater than that with a mask, which is consistent with the theory. The *a*_1_ values of different mask types also have differentiation, which will be further explained.

In summary, the above three features can distinguish whether or not to wear a mask by setting the thresholds appropriately. The effect of a single feature is limited, while the combination of the three features can achieve better results. The decision conditions are *N > N’, SSD > SSD’*, *a*_1_ > *a*_1_’, where *N’, SSD’*, and *a*_1_’ are the thresholds. In our work, *N’* is 2, *SSD’* is 0.05, and *a*_1_’ is 5. To improve the robustness, the voting method is used for the classification. When more than two evaluation conditions are satisfied, it is considered as without a mask. Otherwise, it is determined to be wearing a mask.

**Figure 11:**
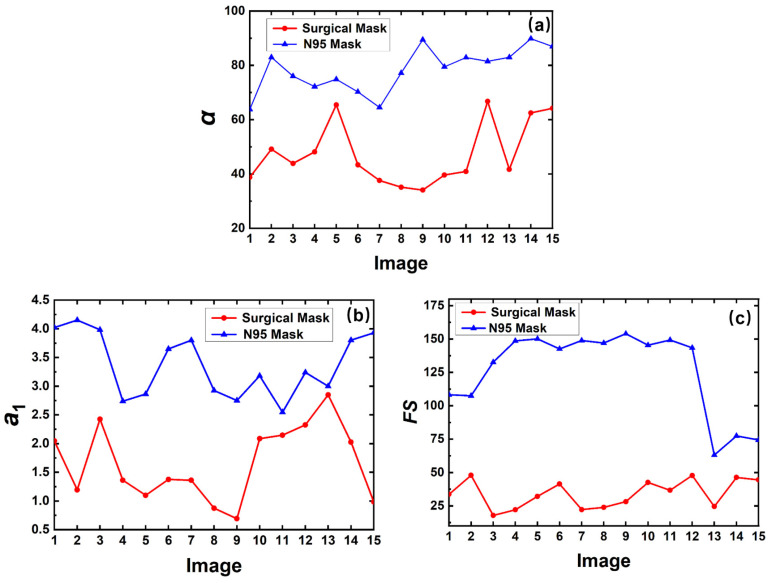
Feature values to identify the mask types captured from 15 images: (**a**) the *α* value, (**b**) the *a*_1_ value, and (**c**) the *FS* value of two curve types.

In the identification process of two mask types, the surgical mask and the N95 mask, three features are extracted from the spatial and frequency curves. Figure 8 shows the features to distinguish the mask types from 15 images. The red and blue curves represent the feature values of the contour curve with the surgical mask and the N95 mask, respectively. The opening angle values of these images are illustrated in Figure 11a. It clearly shows that the opening angle of the contour curve with the N95 mask is greater than that with the surgical mask. However, the opening angle values of two types still have overlapping parts and other features are required to be leveraged together.

The quadratic coefficient *a*_1_ of the partial fitting curve is shown in Figure 11b. Although the opening angle of the contour curve with an N95 mask is larger than that with a surgical mask, the quadratic coefficient of the partial fitting curve of an N95 mask is smaller, because the chosen part of the N95 mask is sharper together with a more stable shape.

The frequency curve is obtained by the Fourier transform after the quadratic fitting of the contour curve. The area of the frequency curve of the N95 mask is larger than that of the surgical mask in Figure 11c. 

The above three features can distinguish whether or not to wear a mask by setting the thresholds appropriately. Due to different wearing habits and soft mask materials, the feature values have fluctuations, and the effect of a single feature is limited. The combination of these three features can achieve better results, and the voting method is also used to classify the mask types. The decision conditions are *α < α’*, *a*_1_ < *a*_1_’, *FS < FS’*, where *α’*, *a*_1_’, and *FS’* are thresholds. In our work, *α’* is 70, *a*_1_’ is 2.5, and *FS’* is 85. When two or more threshold conditions are satisfied, it is determined that the person is wearing a surgical mask. Otherwise, it is judged as an N95 mask.

The algorithm is evaluated from many aspects based on our data. The results can be classified into three categories: *TP* (true positive) means that the results are the same as the real conditions; *FP* (false positive) means samples in the detected category are inconsistent with the real category; and *FN* (false negative) means that the object is detected as the opposite category. Precision is used to measure the proportion of the real cases (*TP*) among all the positive cases (*TP* + *FP*) detected by the algorithm, as shown in Equation (6).

(6) $$Precision=\frac{TP}{TP+FP}$$

Recall is the proportion of detected real cases in all positive cases, which represents the ability of the algorithm to detect the real cases, calculated as follows:

(7) $$Recall=\frac{TP}{TP+FN}$$

Due to the trade-off between the precision and recall, the *F*_1_ score combines them into a single indicator, defined by the harmonic average, as shown in Equation (8).

(8) $$F_{1}=2\times \frac{P\times R}{P+R}=\frac{TP}{TP+\frac{FN+FP}{2}},$$

where *P* and *R* represent precision and recall, respectively. If the *F*_1_ score is higher, the test of the algorithm will be more effective.

A total of 369 depth images were captured, including evenly distributed categories: without masks, with surgical masks, and with N95 masks, to test the algorithm. These images were taken by 3 women aged from 23 to 25 over a 1-week timeframe. In order to enrich the sample diversity, the images were taken for people with and without glasses. In addition, it also includes different hairstyles, such as long, straight hair and a ponytail. In the proposed algorithm, there are two classifications: one is whether or not to wear mask and the other is the mask types. The precision, recall, and *F*_1_ score are calculated for the two cases, respectively. For the classification of whether or not to wear mask, the results are listed in Table 1.

Although the recall of those without a mask is larger than the recall of those with a mask, the precision and *F*_1_ score of with a mask are larger than that of without a mask. As displayed in Table 1, most images without a mask can be detected correctly. Because of the soft material, surgical masks are more likely to produce wrinkles or reflect the undulation of the face. Therefore, most of the images incorrectly identified as without a mask are actually images with surgical masks.

To evaluate the confusion between the surgical mask and the N95 mask, the classification results on the mask types are shown in Table 2. Since the step is performed after detecting whether or not to wear a mask, the classification of mask types relies on the image detected as with a mask. These two kinds of masks have similar contour curves and feature values, which leads to the drop in accuracy. In addition, the precision and recall of the two types are in a restrictive relation, respectively, and have close *F*_1_ scores.

The system runs on a regular laptop (Intel Core AMD Ryzen 7 6800H CPU running at 3.2 GHz, and 16 GB of RAM) and MATLAB is used for image processing. The average processing time of the algorithm is 32 ms, which corresponds to 31.55 FPS.

Table 3 presents a comparison of this proposal with the other works. The accuracy of our work is expressed by the average precision. At present, there is less related work to recognize the face mask based on the depth image; most works are based on the RGB images. However, compared with the method based on the RGB image, the depth image captured from the low-cost ToF camera can be less affected by the environment light. Therefore, our accuracy has certain advantages compared with those works. The work [14] considered the RGB data under dark condition; they introduced additional resources to train the network, and the improved accuracy rate in the dark condition was 77.9%. Compared with this work, our proposed method is unaffected by light and the average accuracy can reach 96.9%. In addition, we have also classified the mask types, which are not involved in some works. It can be seen from Jiang et al. [12] that the introduction of more classification situations will affect the accuracy of the method. In the case of sufficient lighting and good image quality, our ToF-camera-based method has close accuracy with the other works of mask recognition based on the RGB images. Most RGB-camera-based works with high identification accuracy are based on mature deep-learning methods. However, compared with most network-based works, our method is more interpretable and with lower computational costs.

## 4. Conclusions and Discussion

We propose a method to identify whether a person is wearing mask, which can further classify the mask types, such as the surgical mask and the N95 mask. A ToF camera with a simple system and robust data is used to capture the depth image, especially in dark conditions. The designed method can extract facial contours accurately by using the depth information. Facial contour extraction is essentially the sampling and dimensionality reduction of the depth data, which can reduce the amount of data and improve the recognition speed of the algorithm. Additionally, the spatial and frequency features of the facial contour are further extracted. With the appropriate thresholds and the voting method, it can achieve a 99.21% recall accuracy for images without a mask, and a 94.6% recall accuracy for images with a mask. Through reducing the dimension of the data, our method can reach satisfactory accuracy while quickly identifying.

However, there is certain confusion between the two mask types, and the soft material of the mask is the main reason. Therefore, single-depth information is insufficient. In the future, we hope to combine the depth distance with other information to build a better model. Furthermore, people have different habits of wearing masks. The classification category also needs to be further developed, such as the classification of incorrectly worn masks, the recognizable mask types, and the application scenarios. In practical applications, there will be more complex challenges, such as people’s movement and a complex crowd environment. When an angle formed between the face and the ToF camera, some depth information will be lost for the occluded part of the face. When the angle between the person and the camera is less than 90°, the facial contour curve can be detected by raw-data rotation, which is also our next research focus. Additionally, the work on larger rotation angles and different directions is also under further study. In addition, the data of multiple people and in motion are also being further considered.

## Figures and Tables

**Table 1:** Detection results of whether or not to wear mask.

Category	Precision	Recall	*F*_1_ Score
Without a mask	0.906	0.992	0.947
With a mask	0.996	0.946	0.970

**Table 2:** Detection results of whether or not to wear mask.

Category	Precision	Recall	*F*_1_ Score
With a surgical mask	0.847	0.909	0.877
With an N95 mask	0.910	0.842	0.875

**Table 3:** Comparison of this proposal with other works.

Work	Method	Data	Distinguished Type	Accuracy	Efficiency
Ours	Feature-based	Depth	With/Without	96.9%	31.55 FPS
			Mask Type	87.85%	
Cao et al. [14]	YOLOv4-large	RGB	With/Without	94%	18 FPS
			Night Time	77.9%	
Nagrath et al. [10]	SSDMNV2	RGB	With/Without	92.64%	15.71 FPS
Jiang et al. [12]	SE-YOLOv3	RGB	With/Without/ Correct Wearing	73.7%	15.63 FPS
Walia et al. [7]	YOLOv5	RGB	With/Without	98%	32 FPS
Su et al. [11]	Transfer Learning and Effcient-Yolov3	RGB	With/Without	96.03%	15 FPS
			Mask Type	97.84%	
Yu et al. [8]	YOLO-v4	RGB	With/Without	98.3%	54.57 FPS

## Data Availability

Data underlying the results presented in this paper are not publicly available at this time but may be obtained from the authors upon reasonable request.

## References

[1] Cheng Y., Ma N., Witt C., Rapp S., Wild P.S., Andreae M.O., Pöschl U., Su H. (2021). Face Masks Effectively Limit the Probability of SARS-CoV-2 Transmission. Science.

[2] Mbunge E., Chitungo I., Dzinamarira T. (2021). Unbundling the Significance of Cognitive Robots and Drones Deployed to Tackle COVID-19 Pandemic: A Rapid Review to Unpack Emerging Opportunities to Improve Healthcare in Sub-Saharan Africa. Cogn. Robot..

[3] Goar V., Sharma A., Yadav N.S., Chowdhury S., Hu Y.-C. (2022). IoT-Based Smart Mask Protection against the Waves of COVID-19. J. Ambient Intell. Hum. Comput..

[4] Rahim M.S.A., Yakub F., Hanapiah A.R.M., Rashid M.Z.A. Development of Low-Cost Thermal Scanner and Mask Detection for Covid-19. Proceedings of the 2021 60th Annual Conference of the Society of Instrument and Control Engineers of Japan (SICE).

[5] Hussain S., Yu Y., Ayoub M., Khan A., Rehman R., Wahid J.A., Hou W. (2021). IoT and Deep Learning Based Approach for Rapid Screening and Face Mask Detection for Infection Spread Control of COVID-19. Appl. Sci..

[6] Zhao Z.-Q., Zheng P., Xu S., Wu X. (2019). Object Detection with Deep Learning: A Review 2019. IEEE Trans. Neural Netw. Learn. Syst..

[7] Walia I.S., Kumar D., Sharma K., Hemanth J.D., Popescu D.E. (2021). An Integrated Approach for Monitoring Social Distancing and Face Mask Detection Using Stacked ResNet-50 and YOLOv5. Electronics.

[8] Yu J., Zhang W. (2021). Face Mask Wearing Detection Algorithm Based on Improved YOLO-V4. Sensors.

[9] Talahua J.S., Buele J., Calvopiña P., Varela-Aldás J. (2021). Facial Recognition System for People with and without Face Mask in Times of the COVID-19 Pandemic. Sustainability.

[10] Nagrath P., Jain R., Madan A., Arora R., Kataria P., Hemanth J. (2021). SSDMNV2: A Real Time DNN-Based Face Mask Detection System Using Single Shot Multibox Detector and MobileNetV2. Sustain. Cities Soc..

[11] Su X., Gao M., Ren J., Li Y., Dong M., Liu X. (2022). Face Mask Detection and Classification via Deep Transfer Learning. Multimed. Tools Appl..

[12] Jiang X., Gao T., Zhu Z., Zhao Y. (2021). Real-Time Face Mask Detection Method Based on YOLOv3. Electronics.

[13] Kumar A., Kaur A., Kumar M. (2019). Face Detection Techniques: A Review. Artif. Intell. Rev..

[14] Cao Z., Shao M., Xu L., Mu S., Qu H. (2020). MaskHunter: Real-time Object Detection of Face Masks during the COVID-19 Pandemic. IET Image Process.

[15] Daneshmand M., Helmi A., Avots E., Noroozi F., Alisinanoglu F., Arslan H.S., Gorbova J., Haamer R.E., Ozcinar C., Anbarjafari G. (2018). 3D Scanning: A Comprehensive Survey. arXiv.

[16] Sansoni G., Trebeschi M., Docchio F. (2009). State-of-The-Art and Applications of 3D Imaging Sensors in Industry, Cultural Heritage, Medicine, and Criminal Investigation. Sensors.

[17] Zhang S. (2018). High-Speed 3D Shape Measurement with Structured Light Methods: A Review. Opt. Lasers Eng..

[18] He Y., Chen S. (2019). Recent Advances in 3D Data Acquisition and Processing by Time-of-Flight Camera. IEEE Access.

[19] Cippitelli E., Fioranelli F., Gambi E., Spinsante S. (2017). Radar and RGB-Depth Sensors for Fall Detection: A Review. IEEE Sensors J..

[20] Zanuttigh P., Marin G., Dal M.C., Dominio F., Minto L., Cortelazzo G.M. (2016). Time-of-Flight and Structured Light Depth Cameras: Technology and Applications.

[21] Horaud R., Hansard M., Evangelidis G., Ménier C. (2016). An Overview of Depth Cameras and Range Scanners Based on Time-of-Flight Technologies. Mach. Vis. Appl..

[22] Aggarwal J.K., Xia L. (2014). Human Activity Recognition from 3D Data: A Review. Pattern Recognit. Lett..

[23] Xu T., An D., Wang Z., Jiang S., Meng C., Zhang Y., Wang Q., Pan Z., Yue Y. (2020). 3D Joints Estimation of the Human Body in Single-Frame Point Cloud. IEEE Access.

[24] Bae J.-H., Jo H., Kim D.-W., Song J.-B. Grasping System for Industrial Application Using Point Cloud-Based Clustering. Proceedings of the 2020 20th International Conference on Control, Automation and Systems (ICCAS).

[25] Zengeler N., Kopinski T., Handmann U. (2018). Hand Gesture Recognition in Automotive Human–Machine Interaction Using Depth Cameras. Sensors.

[26] Gai J., Xiang L., Tang L. (2021). Using a Depth Camera for Crop Row Detection and Mapping for Under-Canopy Navigation of Agricultural Robotic Vehicle. Comput. Electron. Agric..

[27] Eric N., Jang J.-W. Kinect Depth Sensor for Computer Vision Applications in Autonomous Vehicles. Proceedings of the 2017 Ninth International Conference on Ubiquitous and Future Networks (ICUFN).

[28] Procházka A., Schätz M., Vyšata O., Vališ M. (2016). Microsoft Kinect Visual and Depth Sensors for Breathing and Heart Rate Analysis. Sensors.

[29] Luna C.A., Losada-Gutiérrez C., Fuentes-Jiménez D., Mazo M. (2021). Fast Heuristic Method to Detect People in Frontal Depth Images. Expert Syst. Appl..

[30] Das S., Sarkar S., Das A., Das S., Chakraborty P., Sarkar J. (2021). A Comprehensive Review of Various Categories of Face Masks Resistant to Covid-19. Clin. Epidemiol. Glob. Health.

